# Comparison of state-of-the-art deep learning architectures for detection of freezing of gait in Parkinson’s disease

**DOI:** 10.3389/fneur.2023.1306129

**Published:** 2023-12-21

**Authors:** Emilie Charlotte Klaver, Irene B. Heijink, Gianluigi Silvestri, Jeroen P. P. van Vugt, Sabine Janssen, Jorik Nonnekes, Richard J. A. van Wezel, Marleen C. Tjepkema-Cloostermans

**Affiliations:** ^1^Department of Neurology and Clinical Neurophysiology, Medical Spectrum Twente, Enschede, Netherlands; ^2^Department of Neurobiology, Donders Institute for Brain, Cognition and Behaviour, Radboud University, Nijmegen, Netherlands; ^3^OnePlanet Research Center imec-the Netherlands, Wageningen, Netherlands; ^4^Department of Rehabilitation, Centre of Expertise for Parkinson and Movement Disorders, Donders Institute for Brain, Cognition and Behaviour, Radboud University Medical Centre, Nijmegen, Netherlands; ^5^Department of Biomedical Signals and Systems, MedTech Centre, University of Twente, Enschede, Netherlands; ^6^Department of Neurology, Anna Hospital, Geldrop, Netherlands; ^7^Department of Rehabilitation, Sint Maartenskliniek, Nijmegen, Netherlands; ^8^Department of Clinical Neurophysiology, MedTech Centre, University of Twente, Enschede, Netherlands

**Keywords:** Parkinson’s disease, freezing of gait, wearable sensors, accelerometer, convolutional neural network, InceptionTime, MiniRocket, deep learning

## Abstract

**Introduction:**

Freezing of gait (FOG) is one of the most debilitating motor symptoms experienced by patients with Parkinson’s disease (PD). FOG detection is possible using acceleration data from wearable sensors, and a convolutional neural network (CNN) is often used to determine the presence of FOG epochs. We compared the performance of a standard CNN for the detection of FOG with two more complex networks, which are well suited for time series data, the MiniRocket and the InceptionTime.

**Methods:**

We combined acceleration data of people with PD across four studies. The final data set was split into a training (80%) and hold-out test (20%) set. A fifth study was included as an unseen test set. The data were windowed (2 s) and five-fold cross-validation was applied. The CNN, MiniRocket, and InceptionTime models were evaluated using a receiver operating characteristic (ROC) curve and its area under the curve (AUC). Multiple sensor configurations were evaluated for the best model. The geometric mean was subsequently calculated to select the optimal threshold. The selected model and threshold were evaluated on the hold-out and unseen test set.

**Results:**

A total of 70 participants (23.7 h, 9% FOG) were included in this study for training and testing, and in addition, 10 participants provided an unseen test set (2.4 h, 11% FOG). The CNN performed best (AUC = 0.86) in comparison to the InceptionTime (AUC = 0.82) and MiniRocket (AUC = 0.76) models. For the CNN, we found a similar performance for a seven-sensor configuration (lumbar, upper and lower legs and feet; AUC = 0.86), six-sensor configuration (upper and lower legs and feet; AUC = 0.87), and two-sensor configuration (lower legs; AUC = 0.86). The optimal threshold of 0.45 resulted in a sensitivity of 77% and a specificity of 58% for the hold-out set (AUC = 0.72), and a sensitivity of 85% and a specificity of 68% for the unseen test set (AUC = 0.90).

**Conclusion:**

We confirmed that deep learning can be used to detect FOG in a large, heterogeneous dataset. The CNN model outperformed more complex networks. This model could be employed in future personalized interventions, with the ultimate goal of using automated FOG detection to trigger real-time cues to alleviate FOG in daily life.

## 1 Introduction

Freezing of gait (FOG) is one of the most debilitating symptoms of Parkinson’s disease (PD). During freezing, people have the feeling that their feet are glued to the floor. FOG occurs in 20–60% of people in later stages of PD (1, 2) and it highly impairs mobility, causes falls, and reduces the quality of life (2). FOG can manifest in phenotypes such as trembling in place or walking with small shuffling steps. A third, less common phenotype of FOG is akinesia, in which the person cannot move their legs at all. FOG can be evoked by various situational triggers, such as turning, dual-tasking, walking through a doorway, or starting a movement (3).

The development of objective FOG detection is very relevant (4), as it could be used to provide for (as well as evaluate the effect of) personalized interventions such as cueing (3, 5). The current gold standard for analyzing FOG is visual video annotation by independent experienced raters (6). However, this is very time-consuming and not desirable nor feasible in daily living situations, because of privacy issues, nor could it be used for providing interventions in real time. Inertial measurement units (IMUs), containing an accelerometer and a gyroscope, could overcome this problem as they allow for objective analysis of movement. A recent review summarized the numerous studies investigating automated FOG detection based on IMU data by supervised and unsupervised learning techniques (7). Despite extensive research, the investigated datasets are often relatively small (between 4 and 34 participants) (7).

More recent work demonstrated the potential of FOG detection in a large dataset; a convolutional neural network (CNN) was able to detect FOG in 59 freezers, with 79.6% sensitivity and a specificity of 93.3% (8). The ideal algorithm is, however, yet to be found (3).

### 1.1 Related work

Several deep learning techniques have been applied to detect FOG in IMU data, such as recurrent neural networks (RNNs), besides a CNN (7). A recent review by Sigcha et al. described the use of wearable sensors and deep learning for the monitoring and diagnosis of PD (9). They found fifteen studies describing the use of wearables and deep learning for FOG detection. The studies had a large range in sample sizes, from 7 to 63 people with PD. A total of 14 out of 15 studies used acceleration data for FOG detection, of which 6 used acceleration data in combination with gyroscope data. In all, 10 out of 15 studies used a CNN to detect FOG (9), of which 4 studies used raw data as input for the CNN (10–13).

Sample sizes remained small in these 4 studies, from 7 to 11 participants. Several sensor locations were used in the studies, such as the lumbar, thigh, ankles, and wrists. CNNs consisted of 2–4 convolutional layers, often paired with batch normalization, pooling, and drop-out layers. All four studies used a leave-one-subject-out (LOSO) validation and achieved a sensitivity of 63–95%, specificity of 73–99%, and area under the curve (AUC) of 0.83–0.93 (10–13). Only CNNs, long-short-term memory networks, and a convolutional denoising autoencoder were described in the review (14), which leaves an opportunity for better FOG classification for deeper models, given more data is provided.

Another review (15) described machine learning results of more complex models analyzing time series data, such as IMU data. The results suggested that better performance could be achieved by implementing more complex networks instead of a CNN, however, not all discussed networks have been tested on FOG data (15). The networks with the greatest potential were the Rocket and InceptionTime, which outperformed other networks on time series classification (15). Rocket is further succeeded by the MiniRocket, which is a fast data mining classifier (16). InceptionTime is an ensemble of deep CNN models (17), and while Inception-based networks have previously been used to detect FOG with high accuracy (93.5%) (18), high sensitivity (98%), and high specificity (99%) (19), the InceptionTime model specifically has not been tested yet for FOG detection. The MiniRocket has also not been tested to detect FOG on IMU data (7, 15).

Given the previous results on other IMU datasets, we hypothesized that MiniRocket and InceptionTime models would result in higher accuracy in detecting FOG in comparison to a standard CNN. The objective of this study was to compare those three architectures for the detection of FOG in a large dataset of 70 people. To create this large dataset we combined IMU data across four previously conducted studies (20–23). These studies all focused on people with PD who experienced FOG and who performed various gait tasks, using the same motion capture equipment. We also evaluated the effect of the amount and location of worn IMUs on the sensitivity and specificity of FOG detection in order to facilitate FOG detection methods during daily living. The final model was tested on a hold-out test set and a separate, independent dataset, to evaluate its generalizability to participants unseen by the model.

## 2 Methods

### 2.1 Data

Data from four previous studies were combined into one dataset (20–23) and used for training and validation. These four studies included people with PD who experienced regular FOG. Regular FOG was defined as experiencing it minimally twice a day (20–23). Participants were measured in both the ON and OFF dopaminergic states. Duplicate participants between studies were identified and their data was coupled. Participants were split into a training (80%) and a hold-out test set (20%) using Scikit-Learn and stratified by the occurrence of FOG (24). An additional test set of 10 people with PD and FOG was obtained from unpublished work from Cockx et al. (25), to test whether the final model could generalize to unseen patients and movements. Some studies included healthy controls, but they were disregarded for this analysis.

All data consisted of IMU data obtained by the MVN Awinda motion capture system (Xsens, Enschede, the Netherlands) running MVN Analyze software (26). The IMUs had an accelerometer range of ±160 m/s^2^ (16g) and a sensitivity of 7.8 mg/LSB (26). Specifically, the data consisted of full body measurements, comprising the seven sensor locations of interest to this work; the lumbar region, upper legs, lower legs, and both feet (20–23). The sample frequency was 60 Hz for full body data, and the sample frequency was 100 Hz if only lower body measurements were conducted. The data contained several gait tasks such as walking straight forward, walking through a narrow passage, turning on the spot (360-degree turns), and walking a gait trajectory including 360-degree turns. The unseen test set contained walking straight forward, walking through a narrow passage, turning on the spot, and voluntary stopping.

All gait tasks were recorded by video and annotated offline for FOG. A FOG episode was defined as a “brief, episodic absence or marked reduction of forward progression of the feet despite the intention to walk” (27). The number of FOG episodes and duration were scored from video recordings by two independent and experienced raters. The studies from Janssen et al. reported a high degree of consensus in all three studies for both the number of episodes and total duration of FOG episodes per participant (20–22). In the work of Klaver et al. (23) the degree of consensus was not reported. In all studies, disagreements were discussed until a consensus was reached. Motion and video data were synchronized by two different methods. The studies of Janssen et al. (20–22) synchronized the data by playing a sound signal at the start of motion data acquisition. The sound was used in the video recordings to determine the start of each measurement. Participants in the study from Klaver et al. (23) were instructed to tap with one foot three times prior to each measurement, which was annotated in video data and detected in the motion data for synchronization.

The acceleration data was translated to the local frame. Segments with an acceleration greater than 100 m/s^2^ or an angular velocity greater than 20 rad/s were labeled as artifacts and removed from the analysis. The data was filtered by a zero phase third-order bandpass Butterworth filter (0.3–15 Hz) to remove drift and to retain the full locomotor and FOG frequency spectrum. Data was down-sampled to 60 Hz if standardization of the sampling frequency across studies was required. Next, the data were windowed into two-second epochs with 75% overlap, similar to previous work from another research group (14). The corresponding FOG label was created per window such that if a window contained > = 25% FOG, the window was labeled as FOG. Windows containing <25% FOG were disregarded during training.

### 2.2 Deep learning models

**Figure 1:**
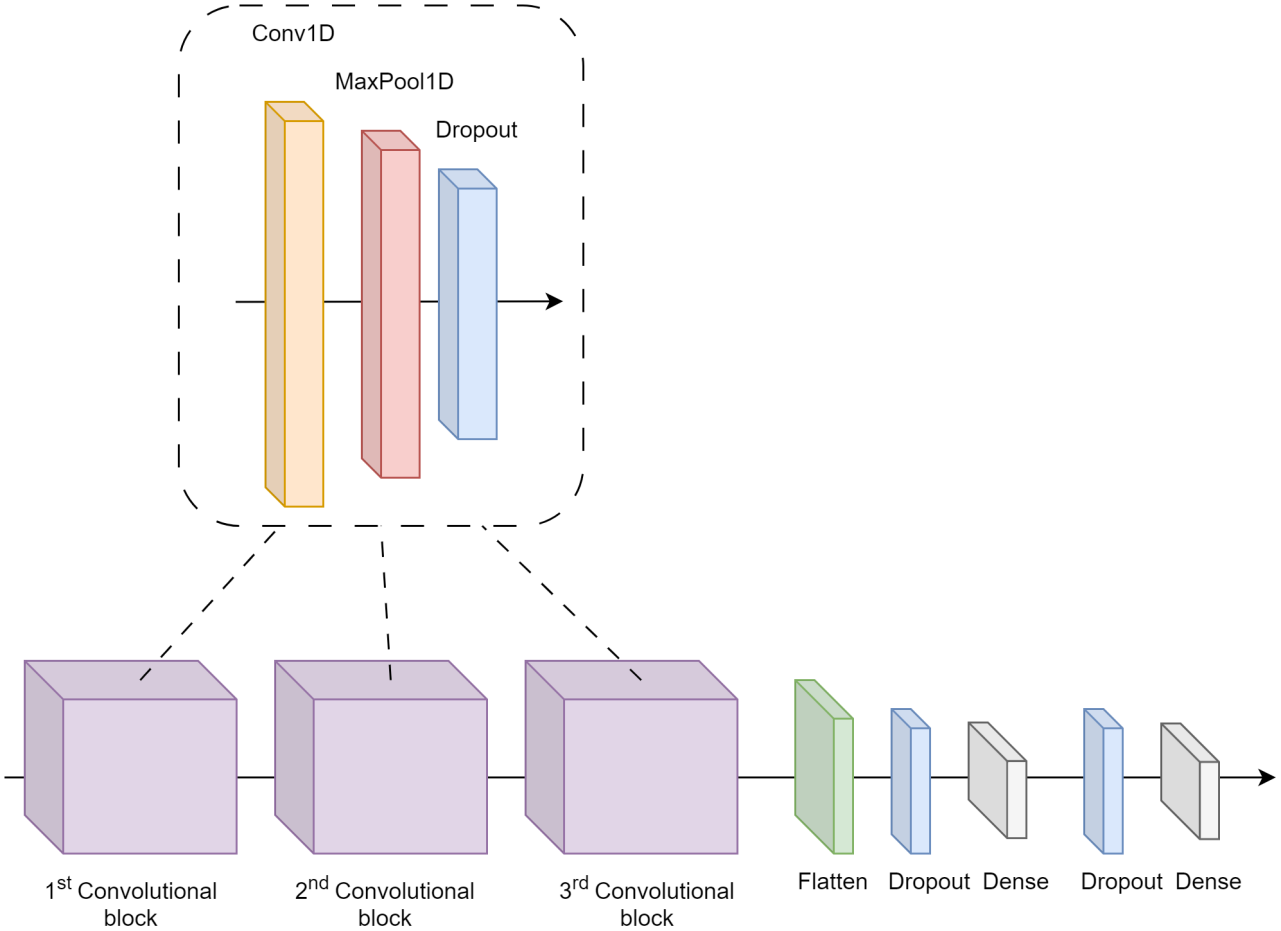
Overview of the CNN architecture.

Three different deep-learning models were trained and tested on the datasets: a CNN, InceptionTime, and MiniRocket. Our code is available at: https://github.com/emilieklaver/FOG_Detection. The CNN consists of three modules; an overview is given in Figure 1. Each module consisted of a convolutional layer with ReLU activation, a max pooling layer of size 2, and a dropout layer with a drop-out rate of 0.2. The kernel size of the first convolutional layer was 7, the subsequent two layers had a kernel size of 3. Class weights were balanced. Data output was flattened after these three modules and then passed through a dropout layer and a dense layer. The first dense layer consisted of 10 neurons with ReLU activation and the final dense layer consisted of 1 neuron with sigmoid activation. The internal parameters were selected *a priori*. The CNN was trained using 30 epochs, a learning rate of 0.001, a batch size of 32, the binary cross-entropy loss function, and 3,727 iterations. We used the AdamW optimizer with a weight decay of 0.001 (28). No early stop conditions were used.

InceptionTime consists of five Inception networks (17). In each inception network, instead of traditional full convolution layers, inception modules are used. A key component of the inception module is a bottleneck layer, which uses sliding filters to reduce the dimensionality of the time series data and the complexity of the model. This makes the model suitable for small datasets, as it reduces overfitting. The model was implemented using TensorFlow and the classification algorithm provided on GitHub (29). Instead of the default kernel sizes of 10, 20, and 40, smaller kernel sizes of 2, 4, and 8 were used to decrease the number of parameters in the network and thus prevent overfitting (17). The InceptionTime was trained with 20 epochs, an initial learning rate of 0.001, and the binary cross-entropy loss function. Class weights were balanced and no weight decay was used.

MiniRocket is the successor of Rocket, which transforms input time series into features to train a linear classifier. MiniRocket maintains the most important characteristics of Rocket; dilation and proportion of positive values pooling (PPV pooling). MiniRocket uses two-valued kernels of a fixed length of 9, which creates a faster classifier. Each kernel has a fixed set of dilations, which is adjusted to the length of the time series. The dilation is limited to a maximum number of dilations of 32 per kernel to keep the transformation efficient. PPV pooling matches a pattern, which reflects the proportion of the input. This results in higher classification accuracy in comparison to other features comparable to global average pooling. As recommended, a logistic regressor was applied as data was >10,000 samples (30). The MiniRocket was implemented using the time series for artificial intelligence (version 0.3.1) in PyTorch (31). We trained the MiniRocket with an initial learning rate of 0.001 for 10 epochs and with a kernel size of 9, no weight decay was used. Logistic loss was used as the loss function and class weights were determined by the model.

The models were trained on the three-directional acceleration data set from all seven sensors of the training set with five-fold cross-validation. Participants were assigned to either the training or validation set per fold to increase the generalizability of the model. The optimal model was identified based on the mean receiving operating characteristic (ROC) curve and the corresponding AUC of the training set. The performance of the optimal model was further analyzed for other sensor configurations, given a total of five scenarios: (1) All seven sensors, namely, the upper leg, lower leg, feet, and lumbar sensors; (2) Six sensors, namely, the upper leg, lower leg, and both feet sensors; (3) Lower leg sensors only; (4) Lumbar sensor only; and (5) Right foot sensor only. When the performance was similar, the optimal model was selected on the minimal amount of sensors. The threshold for the model was calculated by the maximum value of the geometric mean. Finally, the optimal model was tested on the hold-out test set and the unseen test set. The sensitivity, specificity, precision, and F-score were calculated for the threshold selected previously.

## 3 Results

The combined dataset for training, cross-validation, and the hold-out set for testing consisted of 70 uniquely identified people with PD and the unseen test set contained 10 people with PD.

### 3.1 Baseline characteristics

The combined dataset for training, cross-validation, and hold-out testing displayed similar baseline characteristics as the unseen set (Table 1), except for sex (50% men in the unseen test set, whereas the other studies included a higher male population on average: 82%). The combined dataset resulted in 23.7 h of data of which 9% contained FOG. This dataset was split into a training and cross-validation set (*N* = 54) of 20.6 h of data and a hold-out test set of 3.1 h of data (*N* = 16) (Table 2). This resulted in 148,641 samples available for training and cross-validation, and 44,122 samples for testing. The unseen test set consisted of 2.4 h of data, which equates to 34,734 samples, containing 10.8% FOG.

**Table 1:** Baseline characteristics of participants in the training and test sets, given as median (25th-75th percentile).

	Training, validation, and hold-out test sets				Unseen test set
	Janssen et al. (20)	Janssen et al. (22)	Janssen et al. (21)	Klaver et al. (23)	Cockx et al. (25)
No. participants	25	20	16	31	10
Age (years)	72 (65–79)	70.5 (63.5–73)	69 (62–73)	66 (60–74)	69 (66–74)
Sex (% male)	76	85	81	87	50
Disease duration (yrs)	11 (3.0–20.0)	11 (7.5–16.0)	10 (4.0–12.5)	11 (5.0–14.0)	9 (6.0–11.0)
Years since FOG	2 (0.3–12.0)	4 (2.5–6.5)	4 (2.0–10.0)	n.a.	n.a.
Hoehn and Yahr score	2 (2–3)	2 (2–3)	2 (2–3)	2 (2–3)	3 (2–3)
MDS-UPDRS III ON	34 (10–61)	40 (32–48)	38 (28–44)	38 (29–46)	n.a.
MDS-UPDRS III OFF	n.a.	n.a.	n.a.	51 (47–62)	50 (28–53)
FOGQ	n.a.	n.a.	n.a.	15 (13–18)	n.a.
N-FOGQ	18 (8–28)	21 (16–25)	18 (15–21)	n.a.	19 (16–21)
MMSE	28 (19–30)	29 (27–30)	29 (28–30)	28 (26–30)	n.a.
FAB	14 (5–26)	16 (15–17)	17 (16–18)	17 (14–18)	n.a.
MoCA	n.a.	n.a.	n.a.	n.a.	28 (26–28)
TMT B-A	n.a.	n.a.	n.a.	n.a.	42 (29–56)

MDS-UPDRS III, Movement Disorders Society Unified Parkinson’s Disease Rating Scale part III; N-FOGQ, New Freezing of Gait Questionnaire (range 0–28); FOGQ, Freezing of Gait Questionnaire (range 0–24); MMSE, mini-mental state examination (range 0–30); FAB, Frontal Assessment Battery (range 0–18); MoCA, Montreal Cognitive Assessment; TMT B-A, Trail Making Test B-A; ON, ‘on’ dopaminergic state; OFF,‘off’ dopaminergic state.

**Table 2:** Characteristics of the separate datasets.

	Training and validation set	Hold-out test set	Unseen test set
Participants	54	16	10
Signal duration (hours)	20.6	3.1	2.4
Amount of samples	148,641	44,122	34,734
FOG	8.50%	12.50%	10.80%
Amount of FOG episodes	966	279	190

### 3.2 Performance in the training set

**Figure 2:**
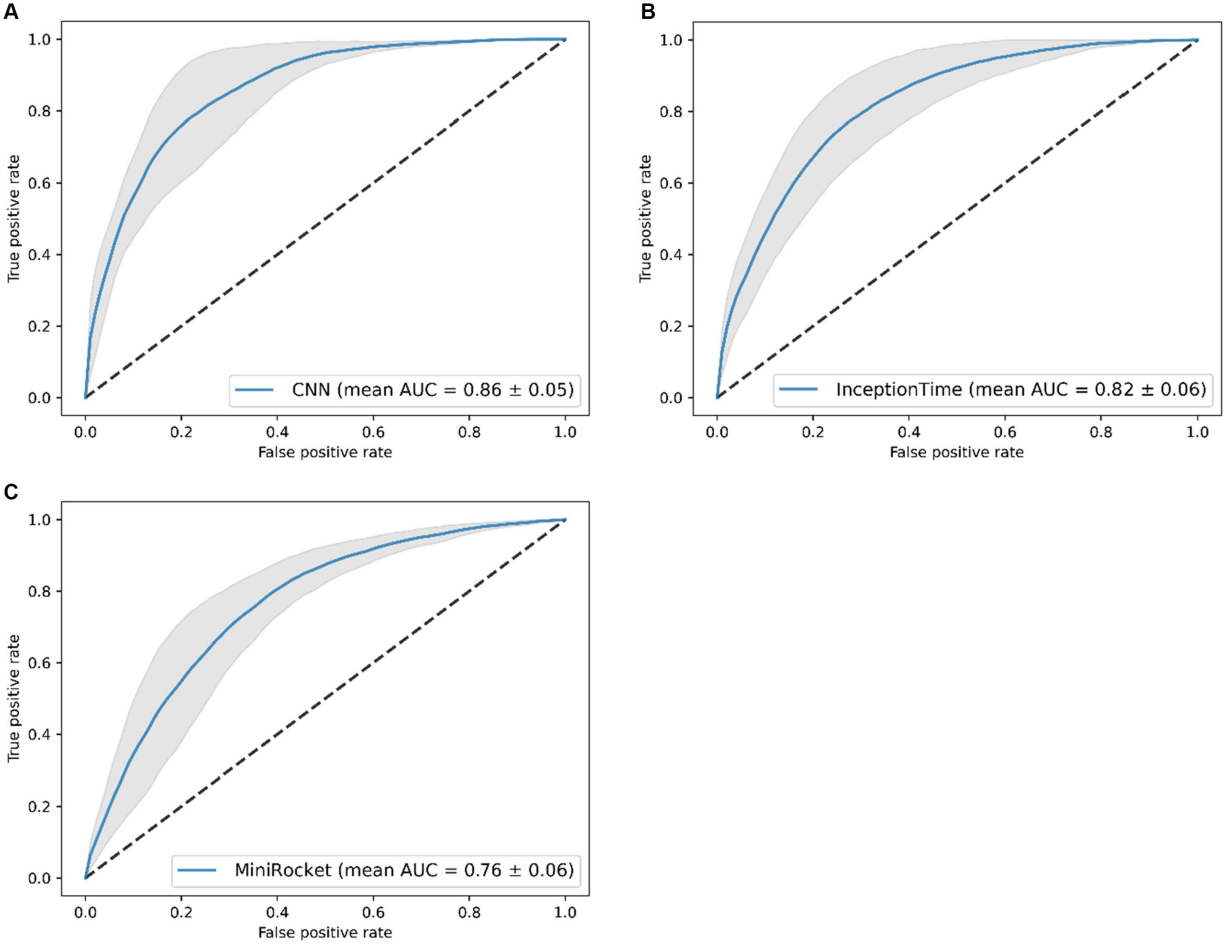
Receiver operating characteristics of the classification models (mean area under the curve (AUC) of five folds ± standard deviation), results of the five-fold cross-validation. Results of: **(A)** the CNN, **(B)** the InceptionTime, and **(C)** the MiniRocket.

**Figure 3:**
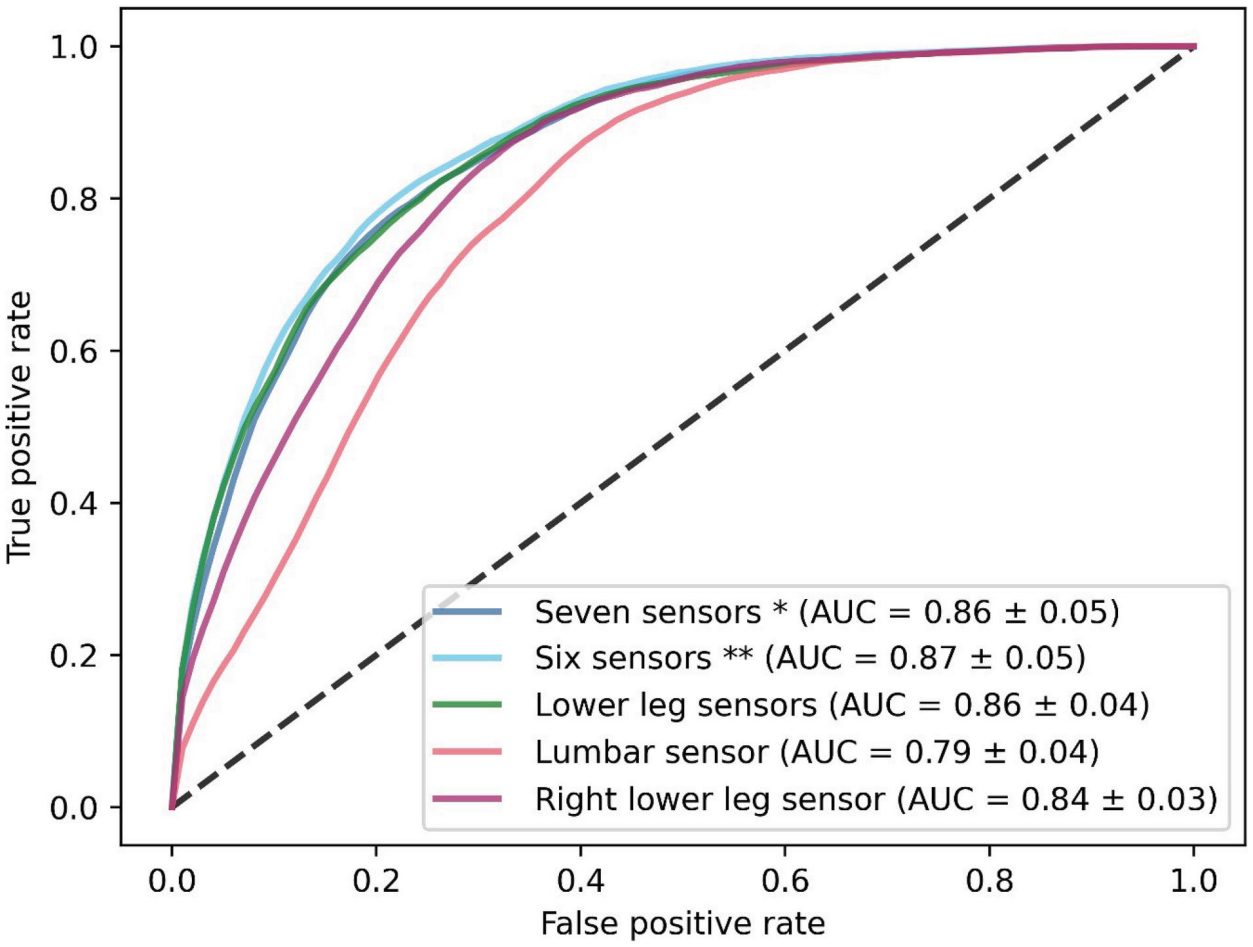
Receiver operating characteristics of the CNN with five different sensor configurations. Results are shown as the mean of the five-fold cross-validation. *Seven sensors: lumbar, upper legs, lower legs, and feet sensors. **Six sensors: upper legs, lower legs, and feet sensors.

The performance of the CNN was superior to the InceptionTime and MiniRocket during training, with a mean AUC of 0.86 [with a standard deviation (SD) of ±0.05] (Figure 2). The six-sensor (upper legs, lower legs, and feet) configuration performed best of all sensor configurations, with a mean AUC of 0.87 (SD ± 0.05). A similar performance was found for the lower leg sensors [AUC of 0.86 (SD ± 0.05)]. Thus, this sensor configuration was considered to be the optimal configuration, as it minimizes the amount of sensors needed. In contrast, we found that for the one-sensor configurations, namely, the lumbar sensor [AUC of 0.79 (SD ± 0.04)] and the right foot sensor [AUC of 0.84 (SD ± 0.03)], performance was poorer in comparison to multiple sensor configurations (Figure 3). The geometric mean (g-mean) was 0.875, with a corresponding threshold for the lower legs configuration of 0.45.

### 3.3 Performance in the hold-out and unseen test sets

**Figure 4:**
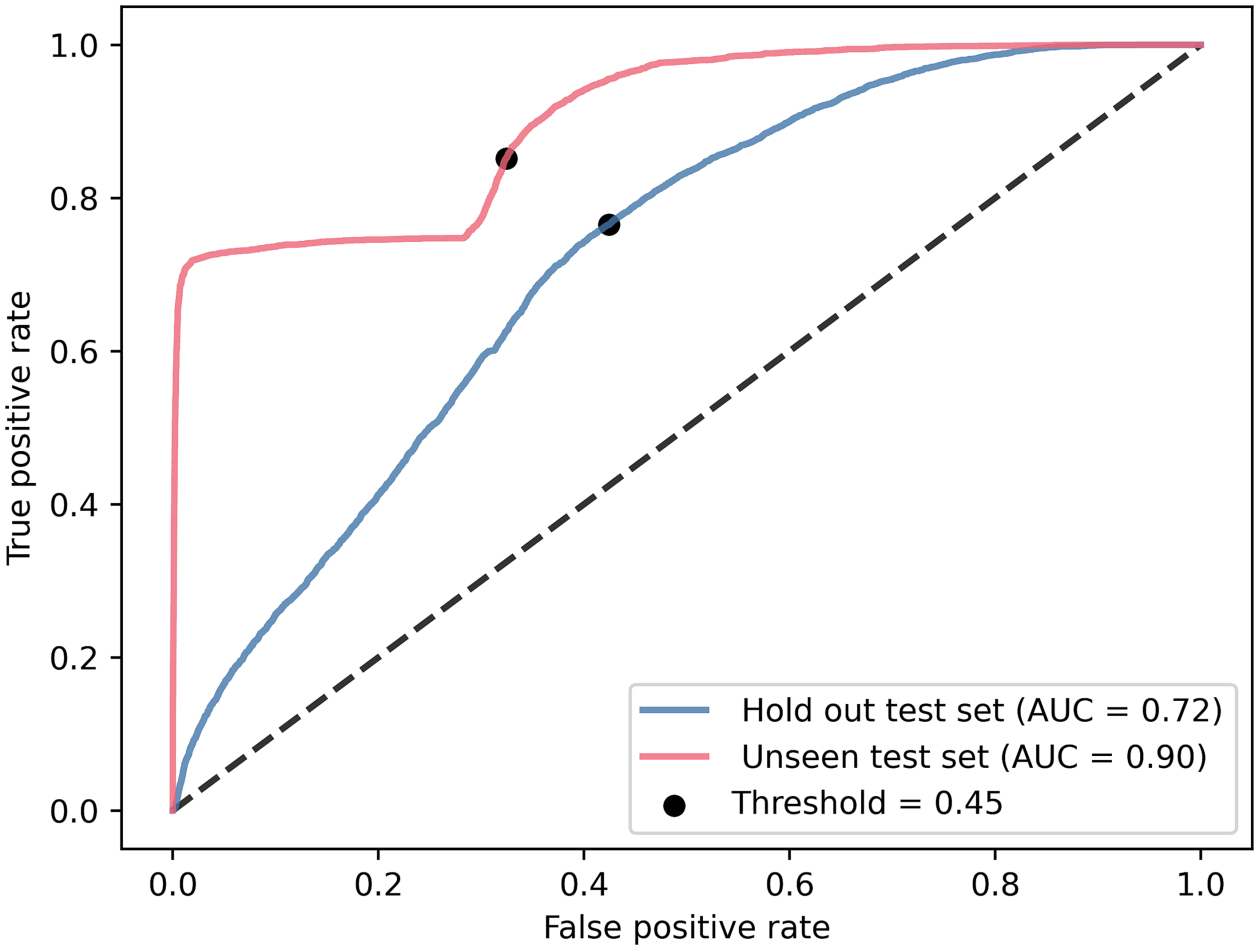
Receiver operating characteristics of the lower legs CNN tested on the hold-out test set and unseen test set.

Evaluation of the optimal model (CNN with IMU data of both lower legs) on our hold-out set resulted in an AUC of 0.72 and for the unseen test set, an AUC of 0.90 (Figure 4). Applying the threshold corresponding to the g-mean resulted in a sensitivity of 77% and a specificity of 58% for the hold-out set (with a precision of 0.21 and F-score of 0.33), and a sensitivity of 85% and a specificity of 68% for the unseen test set (with a precision of 0.26 and F-score of 0.39).

## 4 Discussion

This study aimed to compare two state-of-the-art networks for time series analysis, namely, MiniRocket and InceptionTime, with a CNN for the detection of FOG in IMU data in patients with PD and FOG. We combined multiple datasets to obtain a training set of 70 unique people.

We found that the CNN performed the best based on the AUC, and training the CNN on the lower leg sensors resulted in a mean AUC of 0.86 (SD ± 0.04). We found that the sensitivity (85%) and specificity (68%) of the unseen test set were on par with previous literature, whereas the specificity of the hold-out set (58%) was lower in comparison to the literature (7). However, the corresponding sensitivity of the hold-out set (77%) was also on par with the literature (7). The low precision and low F-score of both the hold-out and unseen test sets are likely the result of an unbalanced data set during training (32) and by the model choice. A more balanced data set could be created by adding more FOG data and by removing participants who did not display any freezing episodes. The current model does not use data augmentation, which could be used to increase the FOG windows to create a more balanced data set. The hyperparameters of the model should be optimized. As data from daily living situations are likely to also be imbalanced, the model could be optimized further by adding a gait detection model prior to freeze detection.

In contrast to our hypothesis, the performance of the InceptionTime and MiniRocket models was inferior to that of the CNN. This could be explained by overfitting, as both the InceptionTime (151,746 trainable parameters) and MiniRocket (9,996 trainable parameters) are more complex than the CNN (7,653 trainable parameters). However, the used training set contained substantially more samples than the largest accelerometer-based training set from the previously mentioned review (15); 148,641 samples versus 316 samples, so it is likely that the difference in performance is not exclusively caused by overfitting. Another explanation could be that the heterogeneity of the data influences the detection of FOG, as the model needs to be able to detect FOG while walking straight forward, walking through a narrow passage, turning on the spot, and walking a gait trajectory including 360-degree turns.

Others have also compared an Inception-based model with other neural networks, such as a CNN and multiple variants of a long short-term memory (LSTM) network (18). They tested the models on the Daphnet freezing of gait dataset (33). The authors found that their iSPLInception network (accuracy of 93.5) performed better in comparison to the standard CNN (accuracy of 93.0), which is in contrast to our work (18). This can be explained by the differences in the used dataset and network structure. Due to the limited amount of data in their dataset, they could not take the subject into account in the split between training, testing, and validation sets. The overlap of subjects between sets might have resulted in an overestimation of the performance of their network. The network is also different from the presented network; in the iSPLInception network, the first input layer is followed by a BatchNorm layer, which is not present in the InceptionTime network utilized in the present study. Kernels in the iSPLInception network have the size of 1, 3, and 5, whereas we used kernels of size 2, 4, and 8 for InceptionTime.

A further expansion of the iSPLInception network has been conducted in which the inSEption and LN-Inception networks were proposed (19). Here, inSEption utilizes the inception module but also includes squeeze and excitation blocks. The LN-inception network differs from the Inception network as it only employs two parallel convolutional operations in order to prevent overfitting. A public dataset was added to the DaphNet dataset, containing the IMU data of 38 people with PD who were turning on the spot (34). The data contained 173 FOG episodes (19, 34). They found remarkable detection of FOG for both models; the LN-Inception had a sensitivity of 97% and specificity of 99%, whereas the inSEption model resulted in a sensitivity of 98% and specificity of 99% (19). This clearly illustrates the potential of an Inception-based model for the detection of FOG. The authors, however, did not describe if participants overlapped between the training and test sets, which could result in high sensitivity and specificity.

The results of our CNN are in line with previous work. In a recent review, Pardoel and Nantel (7) described the results of neural networks of detection of FOG with a sensitivity range of 72.2–99.83% and a specificity range of 48.4–99.96%. As the field of artificial intelligence is moving quickly, after this review in 2019, numerous other research groups reported their work on neural networks for FOG detection. Recent work by Borzì and colleagues, in 2023, used a multi-head CNN and a large dataset of 118 people with PD to detect FOG (8). These authors used three different datasets for their work: the REMPARK, 6MWT, and ADL datasets. The REMPARK dataset consists of 21 people with PD (9.1 h, 93 min of FOG, and 1,058 episodes of FOG) and was used for the initial training. This set was split into a training set, a validation set (*N* = 16), and a hold-out test set (*N* = 5). The 6MWT dataset consists of 38 people with PD (2.4 h, 5.3 min of FOG, and 52 FOG episodes) and was used as an unseen test set. The ADL dataset (5.9 h) contains several gait tasks of 59 people with PD but with no FOG. This dataset was used as an unseen test set to test for false positives (8). Their model resulted in a sensitivity of 87.7% and specificity of 88.3% on their hold-out test set, performing better than the presented work in this paper. However, their hold-out test set was relatively small, containing the data of 5 people, whereas the presented work used a hold-out set of 16 people. Their unseen test set with FOG episodes resulted in a sensitivity of 79.6% and a specificity of 93.3%, which are similar to our results (8). The unseen test set displayed different characteristics when compared to the presented work; and our unseen test set contained data of 10 people, whereas Borzì et al.’s set used the data of 38 people. However, our unseen test contained more data (4.8 h versus 2.4 h) and more freezing episodes (190 episodes versus 52 episodes). Other studies (10–13) that used a CNN with raw acceleration data and smaller sample sizes demonstrated similar results in comparison to the results of the unseen test set. An overview of these studies is given in Table 3.

**Table 3:** Comparison of recent studies and the proposed work.

Study	Number of PD participants	Sensor location (data type)	Validation	Performance	
San-Segunda et al. (11)	11	Lumbar, thigh and ankle (acc)	LOSO	Sensitivity Specificity AUC	95% 73% 0.93
Bikias et al. (12)	11	Wrists (acc, gyro)	LOSO	Sensitivity Specificity	83% 88%
Naghavi and Wade (10)	7	Ankles (acc, gyro)	LOSO	Sensitivity Specificity	63% 99%
O’Day et al. (13)	7	Lumbar and ankles (acc, gyro)	LOSO	AUC	0.83
Borzì et al. (8)	118	Lumbar (acc)	Hold-out	Sensitivity Specificity AUC	88% 88% 0.95
Proposed	Training 54 Hold-out 16 Unseen 10	Lower legs (acc)	Five-fold	*Hold-out* Sensitivity Specificity AUC *Unseen* Sensitivity Specificity AUC	77% 58% 0.72 85%68% 0.90

Studies that used raw data and a CNN to detect FOG were included. acc, acceleration; gyro, gyroscope; LOSO, leave-one-subject-out; AUC, area under the curve.

A strength of our study is that we ensured that the same participants were not distributed amongst both training and test data. This prevented the neural networks from memorizing the characteristics of previously seen patients. Our data included a comprehensive set of gait tasks, such as walking straight ahead, turning, and walking through a narrow passage. This results in a model that is suitable to apply on other datasets and in daily living situations as the training was not individualized or specified for specific gait tasks. This was confirmed by testing the final model on an unseen data set. Surprisingly, we found that our results of the unseen test set outperformed the results of the hold-out set. This may be a result of the types of data from the sets; the unseen test set contains a large amount of walking straight forward, which is also dominant in our training data. Data could unfortunately not be balanced further within gait tasks, as data currently has been randomly assigned by the amount of FOG per participant. Future work could use stratification of the data based on the gait tasks to mitigate this problem. Another strength of this study is the addition of the unseen test set. This data could have been merged with the training set, however, the unseen test set allows us to assess how generalizable the model is.

A drawback of this work, as for any model, is that it is limited by the provided annotations of FOG. The model can only perform as well as the observers do. It is known that observers disagree on the start and end of episodes, which causes a gray area of FOG (35). The joining of several datasets also comes with some limitations. It is likely that the IMU data is not uniform, as slightly different data preprocessing was used as the MVN Analyze software was updated over time. Another limitation of this work is the data selection. Only windows with FOG present over 25% of the time were used for the training set. In real-time data, windows with FOG present below 25% of the time also occur. If the model would be used to activate a medical device to alleviate FOG, then in that case it would be that the algorithm reacts too late to the upcoming FOG episode. This would reduce the effectiveness of the medical device. End-users likely prefer to have the FOG episode detected at the time it occurs. In order to achieve such a goal, a prediction model needs to be developed. Furthermore, data obtained from daily life are likely to contain more complex gait tasks, which could lead to poorer detection of FOG. This could be mitigated by re-training the network with patient-specific at-home data or by implementing a secondary network for gait detection, as previously described by other researchers (36).

In our sensor evaluation, we found the best performance with a six-sensor configuration and found a similar performance for the lower leg sensor configuration. This suggests that not all sensor locations contribute equally to the detection of FOG. We found that one-sensor configurations performed worse in comparison to multiple-sensor configurations. This suggests that the differences between the left and right lower extremity are an important factor in detecting FOG. We also found that the CNN trained on the right lower leg sensors outperformed the lumbar sensor, which holds great promise for integration in future cueing devices. Often, cueing devices are placed at the lower extremities (23, 37, 38) and our results indicate that this is a sufficient measurement site to detect FOG.

In conclusion, we confirmed that deep learning can be used to detect FOG in a large, heterogeneous dataset. In our study, we compared three different models and found that the CNN performed best compared to InceptionTime and MiniRocket. This CNN should be further optimized to create a model that detects FOG with even higher sensitivity and specificity, with the ultimate goal of using automated FOG detection to trigger real-time interventions, such as cues, to alleviate FOG in daily life.

## Data availability statement

The raw data supporting the conclusions of this article will be made available by the authors, without undue reservation.

## Ethics statement

The studies involving humans were approved by Medical Research Ethics Committees United (MEC-U, Nieuwegein). The studies were conducted in accordance with the local legislation and institutional requirements. The participants provided their written informed consent to participate in the studies.

## Author contributions

EK: Conceptualization, Writing – original draft, Writing – review & editing, Formal analysis. IH: Writing – review & editing, Conceptualization, Formal analysis. GS: Writing – review & editing, Methodology. JV: Writing – review & editing, Supervision. SJ: Writing – review & editing. JN: Writing – review & editing, Supervision. RW: Writing – review & editing, Supervision. MT-C: Writing – review & editing, Methodology, Supervision.

## References

[1] Bloem BR Hausdorff JM Visser JE Giladi N. Falls and freezing of gait in Parkinson’s disease: a review of two interconnected, episodic phenomena. Mov Disord. (2004) 19:871–84. doi: 10.1002/mds.20115, PMID: 15300651

[2] Nutt JG Horak FB Bloem BR. Milestones in gait, balance, and falling. Mov Disord. (2011) 26:1166–74. doi: 10.1002/mds.23588, PMID: 21626560

[3] Lewis S Factor S Giladi N Nieuwboer A Nutt J Hallett M. Stepping up to meet the challenge of freezing of gait in Parkinson’s disease. Transl Neurodegener. (2022) 11:23. doi: 10.1186/s40035-022-00298-x, PMID: 35490252 PMC9057060

[4] Moore ST MacDougall HG Ondo WG. Ambulatory monitoring of freezing of gait in Parkinson’s disease. J Neurosci Methods. (2008) 167:340–8. doi: 10.1016/j.jneumeth.2007.08.023 17928063

[5] Nonnekes J Nieuwboer A. Towards personalized rehabilitation for gait impairments in Parkinson’s disease. J Parkinsons Dis. (2018) 8:S101–6. doi: 10.3233/JPD-181464, PMID: 30584154 PMC6311370

[6] Morris TR Cho C Dilda V Shine JM Naismith SL Lewis SJG. A comparison of clinical and objective measures of freezing of gait in Parkinson’s disease. Parkinsonism Relat Disord. (2012) 18:572–7. doi: 10.1016/j.parkreldis.2012.03.001, PMID: 22445248

[7] Pardoel K Nantel L. Wearable-sensor-based detection and prediction of freezing of gait in Parkinson’s disease: a review. Sensors. (2019) 19:5141. doi: 10.3390/s19235141 31771246 PMC6928783

[8] Borzì L Sigcha L Rodríguez-Martín D Olmo G. Real-time detection of freezing of gait in Parkinson’s disease using multi-head convolutional neural networks and a single inertial sensor. Artif Intell Med. (2023) 135:102459. doi: 10.1016/j.artmed.2022.102459, PMID: 36628783

[9] Sigcha L Borzì L Amato F Rechichi I Ramos-Romero C Cárdenas A. Deep learning and wearable sensors for the diagnosis and monitoring of Parkinson’s disease: a systematic review. Expert Syst Appl. (2023) 229:120541. doi: 10.1016/j.eswa.2023.120541

[10] Naghavi N Wade E. Towards real-time prediction of freezing of gait in patients with Parkinson’s disease: a novel deep one-class classifier. IEEE J Biomed Health Inform. (2022) 26:1726–36. doi: 10.1109/JBHI.2021.3103071 34375292

[11] San-Segundo R Torres-Sánchez R Hodgins J De la Torre F. Increasing robustness in the detection of freezing of gait in Parkinson’s disease. Electronics. (2019) 8:119. doi: 10.3390/electronics8020119

[12] Bikias T Iakovakis D Hadjidimitriou S Charisis V Hadjileontiadis LJ. DeepFoG: an IMU-based detection of freezing of gait episodes in Parkinson’s disease patients via deep learning. Front Robot AI. (2021) 8:537384. doi: 10.3389/frobt.2021.537384 34113654 PMC8185568

[13] O’Day J Lee M Seagers K Hoffman S Jih-Schiff A Kidziński Ł. Assessing inertial measurement unit locations for freezing of gait detection and patient preference. J Neuroeng Rehabil. (2022) 19:20. doi: 10.1186/s12984-022-00992-x, PMID: 35152881 PMC8842967

[14] Sigcha L Costa N Pavón I Costa S Arezes P López JM. Deep learning approaches for detecting freezing of gait in Parkinson’s disease patients through on-body acceleration sensors. Sensors. (2020) 20:1895. doi: 10.3390/s20071895, PMID: 32235373 PMC7181252

[15] Ruiz AP Flynn M Large J Middlehurst M Bagnall A. The great multivariate time series classification bake off: a review and experimental evaluation of recent algorithmic advances. Data Min Knowl Discov. (2021) 35:401–49. doi: 10.1007/s10618-020-00727-3, PMID: 33679210 PMC7897627

[16] Dempster A Schmidt DF Webb GI. MiniRocket: a very fast (almost) deterministic transform for time series classification. Proceedings of the 27th ACM SIGKDD Conference on Knowledge Discovery & Data Mining. New York, NY, USA: ACM; (2021). p. 248–257.

[17] Ismail Fawaz H Lucas B Forestier G Pelletier C Schmidt DF Weber J. InceptionTime: finding AlexNet for time series classification. Data Min Knowl Discov. (2020) 34:1936–62. doi: 10.1007/s10618-020-00710-y

[18] Ronald M Poulose A Han DS. iSPLInception: an inception-ResNet deep learning architecture for human activity recognition. IEEE Access. (2021) 9:68985–9001. doi: 10.1109/ACCESS.2021.3078184

[19] Dimoudis D Tsolakis N Magga-Nteve C Meditskos G Vrochidis S Kompatsiaris I. InSEption: a robust mechanism for predicting FoG episodes in PD patients. Electronics. (2023) 12:2088. doi: 10.3390/electronics12092088

[20] Janssen S Bolte B Nonnekes J Bittner M Bloem BR Heida T. Usability of three-dimensional augmented visual cues delivered by smart glasses on (freezing of) gait in Parkinson’s disease. Front Neurol. (2017) 8:279. doi: 10.3389/fneur.2017.00279 28659862 PMC5468397

[21] Janssen S de Ruyter van Steveninck J Salim HS Cockx HM Bloem BR Heida T. The effects of augmented reality visual cues on turning in place in Parkinson’s disease patients with freezing of gait. Front Neurol. (2020) 11:185. doi: 10.3389/fneur.2020.00185 32265826 PMC7105859

[22] Janssen S Heijs JJA van der Meijs W Nonnekes J Bittner M Dorresteijn LDA. Validation of the auditory stroop task to increase cognitive load in walking tasks in healthy elderly and persons with Parkinson’s disease. PLoS One. (2019) 14:e0220735. doi: 10.1371/journal.pone.0220735 31386695 PMC6684087

[23] Klaver EC van Vugt JPP Bloem BR van Wezel RJA Nonnekes J Tjepkema-Cloostermans MC. Good vibrations: tactile cueing for freezing of gait in Parkinson’s disease. J Neurol. (2023) 270:3424–32. doi: 10.1007/s00415-023-11663-9, PMID: 36944760 PMC10267272

[24] Pedregosa F Varoquaux G Gramfort A Michel V Thirion B Grisel O. Scikit-learn: machine learning in Python. J Mach Learn Res. (2011) 12:2825–30.

[25] Cockx HM Oostenveld R RYA Flórez Bloem BR Cameron IGM van Wezel RJA. Freezing of gait in Parkinson’s disease is related to imbalanced stopping-related cortical activity. Manuscript in Preparation. (2023). Retrieved from osf.io/x5a2t 10.1093/braincomms/fcae259 PMC11369826 39229492

[26] Xsens (2018). Available at: https://www.xsens.com/products/

[27] Nutt JG Bloem BR Giladi N Hallett M Horak FB Nieuwboer A. Freezing of gait: moving forward on a mysterious clinical phenomenon. Lancet Neurol. (2011) 10:734–44. doi: 10.1016/S1474-4422(11)70143-0, PMID: 21777828 PMC7293393

[28] Loshchilov I Hutter F. Decoupled weight decay regularization. arXiv. (2017). doi: 10.48550/arXiv.1711.05101

[29] Ismail Fawaz H. InceptionTime. Available at: https://github.com/hfawaz/InceptionTime (Accessed June 2022).

[30] Dempster A Petitjean F Webb GI. ROCKET: exceptionally fast and accurate time series classification using random convolutional kernels. Data Min Knowl Disc. (2019) 34:1454–95. doi: 10.1007/s10618-020-00701-z

[31] Dempster A Schmidt DF Webb GI. MiniRocket. Available at: https://github.com/angus924/minirocket (Accessed July 2022)

[32] Zou Q Xie S Lin Z Wu M Ju Y. Finding the best classification threshold in imbalanced classification. Big Data Res. (2016) 5:2–8. doi: 10.1016/j.bdr.2015.12.001

[33] Bächlin M Plotnik M Roggen D Giladi N Hausdorff JM Tröster G. A wearable system to assist walking of Parkinson’s disease patients. Methods Inf Med. (2010) 49:88–95. doi: 10.3414/ME09-02-0003 20011807

[34] Ribeiro De Souza C Miao R Ávila De Oliveira J Cristina De Lima-Pardini A Fragoso De Campos D Silva-Batista C. A public data set of videos, inertial measurement unit, and clinical scales of freezing of gait in individuals with Parkinson’s disease during a turning-in-place task. Front Neurosci. (2022) 16:832463. doi: 10.3389/fnins.2022.832463, PMID: 35281510 PMC8904564

[35] Cockx H Klaver E Tjepkema-Cloostermans M van Wezel R Nonnekes J. The gray area of freezing of gait annotation: a guideline and open-source practical tool. Mov Disord Clin Pract. (2022) 9:1099–104. doi: 10.1002/mdc3.13556, PMID: 36339306 PMC9631855

[36] Borzì L Sigcha L Olmo G. Context recognition algorithms for energy-efficient freezing-of-gait detection in Parkinson’s disease. Sensors. (2023) 23:4426. doi: 10.3390/s23094426, PMID: 37177629 PMC10181532

[37] Mikos V Heng CH Tay A Yen SC Chia NSY Koh KML. A wearable, patient-adaptive freezing of gait detection system for biofeedback cueing in Parkinson’s disease. IEEE Trans Biomed Circuits Syst. (2019) 13:503–15. doi: 10.1109/TBCAS.2019.2914253 31056518

[38] Sweeney D Quinlan L Browne P Richardson M Meskell P ÓLaighin G. A technological review of wearable cueing devices addressing freezing of gait in Parkinson’s disease. Sensors. (2019) 19:1277. doi: 10.3390/s19061277 30871253 PMC6470562

